# Breast tumor microenvironment structures are associated with genomic features and clinical outcome

**DOI:** 10.1038/s41588-022-01041-y

**Published:** 2022-04-18

**Authors:** Esther Danenberg, Helen Bardwell, Vito R. T. Zanotelli, Elena Provenzano, Suet-Feung Chin, Oscar M. Rueda, Andrew Green, Emad Rakha, Samuel Aparicio, Ian O. Ellis, Bernd Bodenmiller, Carlos Caldas, H. Raza Ali

**Affiliations:** 1 CRUK Cambridge Institute, University of Cambridge, Cambridge, UK; 2 Department of Quantitative Biomedicine, University of Zurich, Zurich, Switzerland; 3 Institute of Molecular Health Sciences, ETH Zurich, Zurich, Switzerland; 4 Department of Histopathology, Addenbrookes Hospital, Cambridge, UK; 5 MRC Biostatistics Unit, University of Cambridge, Cambridge, UK; 6 Department of Pathology, University of Nottingham, Nottingham, UK; 7 British Columbia Cancer Agency, University of British Columbia, Vancouver, British Columbia Canada

**Keywords:** Breast cancer, Tumour immunology

## Abstract

The functions of the tumor microenvironment (TME) are orchestrated by precise spatial organization of specialized cells, yet little is known about the multicellular structures that form within the TME. Here we systematically mapped TME structures in situ using imaging mass cytometry and multitiered spatial analysis of 693 breast tumors linked to genomic and clinical data. We identified ten recurrent TME structures that varied by vascular content, stromal quiescence versus activation, and leukocyte composition. These TME structures had distinct enrichment patterns among breast cancer subtypes, and some were associated with genomic profiles indicative of immune escape. Regulatory and dysfunctional T cells co-occurred in large ‘suppressed expansion’ structures. These structures were characterized by high cellular diversity, proliferating cells and enrichment for *BRCA1* and *CASP8* mutations and predicted poor outcome in estrogen-receptor-positive disease. The multicellular structures revealed here link conserved spatial organization to local TME function and could improve patient stratification.

## Abstract (web summary)

Imaging mass cytometry profiling of 693 breast tumors identified 10 recurrent tumor microenvironment spatial structures. These structures were enriched in different molecular subtypes and can be associated with genomic profiles and outcomes.

## Main

The breast tumor microenvironment (TME) contains specialized cells that behave in a highly coordinated manner^1,2^. Single-cell analyses have revealed the extent of TME cellular diversity^1,2^ but have not addressed how these cells are organized in space. It is clear, however, that characteristic multicellular spatial organization influences tumor phenotype and treatment response. The presence of tertiary lymphoid structures (TLSs), for example, is associated with response to immunotherapy in melanoma and sarcoma^3–5^. Several methods now make highly multiplexed imaging of tissues feasible, enabling precise cell classification in the context of spatial relationships^6–10^. Use of these techniques to analyze solid tumors has begun to uncover principles that govern TME organization^11–14^. A targeted analysis of TME structures in a breast cancer cohort that is large enough to encompass its characteristic heterogeneity has, however, been lacking.

A related question is whether somatic alterations within tumor cells impact TME organization. Some oncogenic alterations have collateral effects that modify the TME, whereas others confer a fitness advantage to cells subject to local selection pressures such as immune predation. Some somatic mutations, for example, dampen the immune response (IR) by causing immune checkpoint overexpression^15–17^, compromised antigen presentation^18,19^ or aberrant interferon signaling^20^. Somatic alterations could therefore trigger a cascade that changes how cells self-organize.

To investigate the landscape of TME structure in breast cancer and its relationship to genomic features and clinical outcome, we used imaging mass cytometry (IMC)^6^ to generate 37-dimensional images of breast tumors from 693 patients recruited to the METABRIC study for whom clinical and genomic data are available^21,22^. We identified ten recurrent TME structures, including quiescent vascularized stroma and several variants of structures associated with an active IR. These TME structures showed distinct associations with somatic alterations and genomic breast cancer subtypes. Regulatory T cells (T_reg_ cells) and dysfunctional T cells co-occurred in large TME structures with high cellular diversity and proliferative cells, which predicted poor outcome. The curated data are available as a resource that, together with our experimental and analytical approach, pave the way for future work to understand principles of spatial organization in cancer tissues.

## Results

### Enumeration of key TME cell phenotypes in situ

**Fig. 1:**
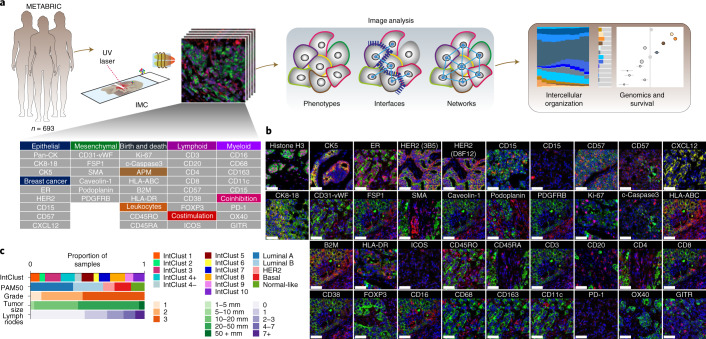
High-dimensional imaging of the breast TME. **a**,Left to right: Antibody panel used for analyses of 693 METABRIC tumors, multitiered image analyses and correlation with genomic and clinical features. **b**, Representative examples of image data (cropped to fit) for proteins of interest (red), pan-cytokeratin (green) and DNA (blue). Scale bars, 50 µm. **c**, Distribution of clinical variables and molecular subtypes among tumors analyzed. IntClust, integrative cluster.

**Extended Data Fig. 1:**
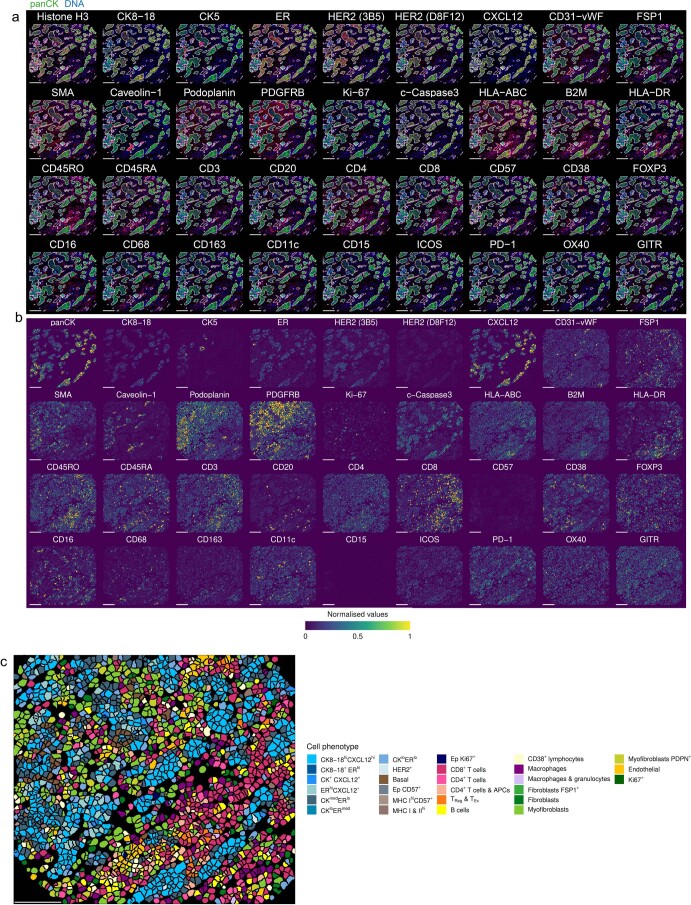
Examples of raw and processed IMC data. **a**, Example of raw IMC data for all channels represented as RGB images. **b**, Processed data for images shown in panel a, depicted as segmented cells colored by mean expression value (data normalized across entire study). **c**, Segmented cell mask for the sample shown in panels a and b colored according to cell phenotype. Scale bars represent 100 µm.

We set out to understand the functional states of the TME in breast cancer. We reasoned intercellular spatial organization would be a strong indicator of function. Using IMC with a panel of antibodies conjugated to isotopically pure rare earth metal reporters (Fig. 1a and Supplementary Table 1), we detected proteins involved in vascular and stromal heterogeneity, antigen presentation and myeloid and lymphoid lineages to identify the cells of the TME and their spatial relationships. Antibodies to immune checkpoint proteins, a costimulatory protein and markers of cell proliferation and apoptosis were included to probe activation states. Antibodies to cytokeratins and canonical breast cancer proteins were included to account for cancer cell heterogeneity. Antibodies were used to label tissue microarray (TMA) slides of breast tumor tissues obtained from 693 patients recruited to the METABRIC study for whom clinical and genomic data are available^21^. The stained slides were analyzed using IMC to generate high-dimensional images (Fig. 1b and Extended Data Fig. 1a). Tumor samples were excised before systemic therapy. The TMAs include tumors of all clinical and molecular subtypes, accounting for intertumor heterogeneity of breast cancer (Fig. 1c).

**Fig. 2:**
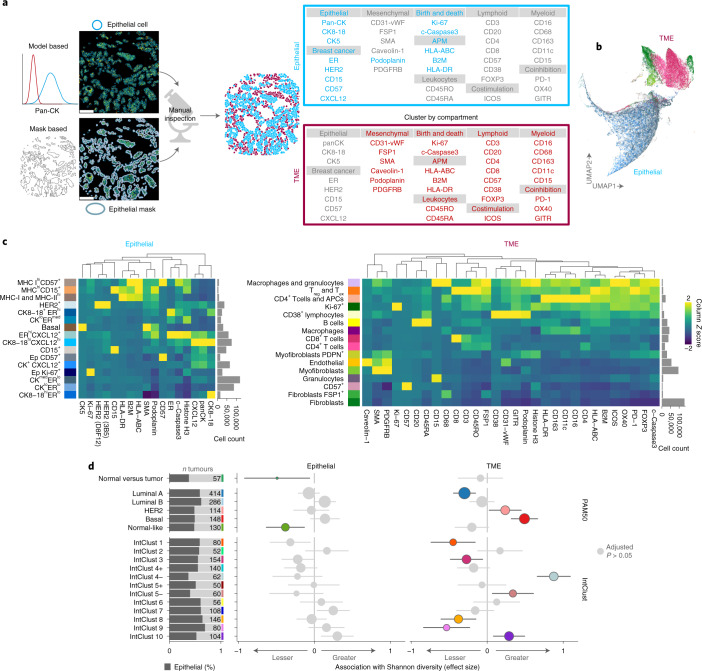
Phenotyping of single cells in situ by IMC. **a**, Analytical workflow for distinguishing epithelial from non-epithelial cells, and proteins used for clustering by compartment. **b**, Scatter plot of uniform manifold approximation and projection (UMAP) dimensions computed from single-cell profiles (random selection of 10% of cells per image per compartment). **c**, Median expression profiles of final cell phenotypes. **d**, Estimates from linear models comparing cell phenotype Shannon diversity of normal versus tumor tissue and among breast cancer molecular subtypes separately for epithelial and TME cells. For comparisons of Shannon diversity in adjacent normal versus tumor tissue, samples were from 655 tumors for epithelial and 660 tumors for TME cells. For comparisons of Shannon diversity between breast cancer molecular subtypes, a total of 546 tumors were included. Values printed on gray bars indicate the number of tumors (or independent normal samples as appropriate) that belong to the group labeled on the *y* axis. Horizontal lines represent 95% confidence intervals. Circles represent point estimates; colored circles indicate estimates associated with an adjusted *P* < 0.05 from two-sided tests for the linear model regression term; *P* values were adjusted for multiple testing using the Benjamini–Hochberg method. Antigen processing machinery, APM; T cell exhaustion, T_ex_.

The key phenotypic distinction between cells in breast tumor tissues is whether they are epithelial or non-epithelial; the latter belong to the TME. To make this distinction most accurately, we adopted two approaches: Gaussian mixture modeling of pan-cytokeratin (pan-CK) expression and capture of cells by a pixel mask trained on all keratins (pan-CK, CK5 and CK8-18) using machine learning. A pathologist then compared the results by manual inspection and selected the best-performing approach for each image (Fig. 2a).

**Extended Data Fig. 2:**
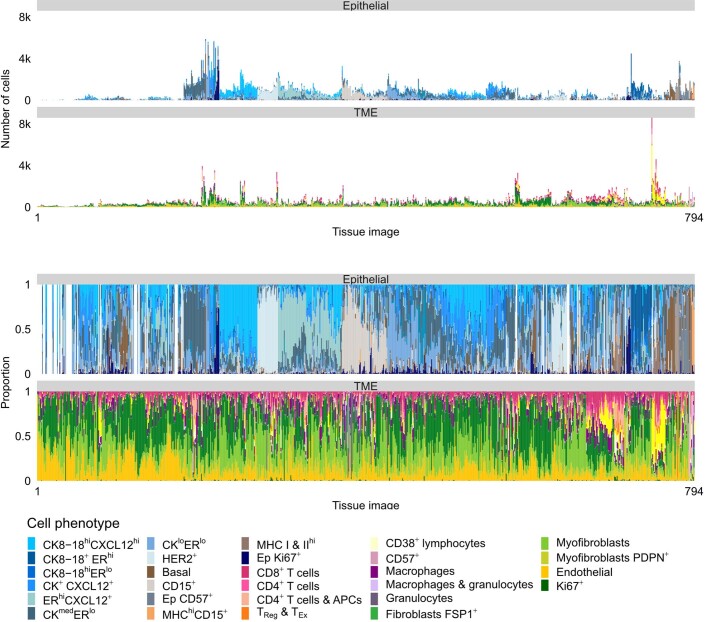
Cell phenotype counts and proportions. Stacked bar plots illustrating the number and proportion of cells by cell phenotype per tissue image. Bars ordered by hierarchical clustering.

Having distinguished cells as epithelial or non-epithelial, we defined cell phenotypes based on the multidimensional protein expression data (Fig. 2b and Extended Data Fig. 1b,c), again combining automated methods with manual curation. Automated single-cell clustering was applied separately within epithelial and non-epithelial compartments. Proteins with exclusive expression in one or the other compartment were selected for clustering (Fig. 2a,b and Extended Data Fig. 2). Cluster expression profiles and cell morphology evaluated by manual inspection were used to assign descriptive labels to clusters, and similar clusters were merged (Fig. 2c). This resulted in 32 cell phenotypes, 16 epithelial and 16 TME. Several epithelial phenotypes were defined by their cytokeratin and hormone receptor profiles, as well as expression of CD15 (ref. ^23^), CD57 (ref. ^24^) and CXCL12; the latter is an ER target^25^. Three phenotypes were distinguished by high expression of antigen-presentation proteins (MHC-I and/or MHC-II).

**Extended Data Fig. 3:**
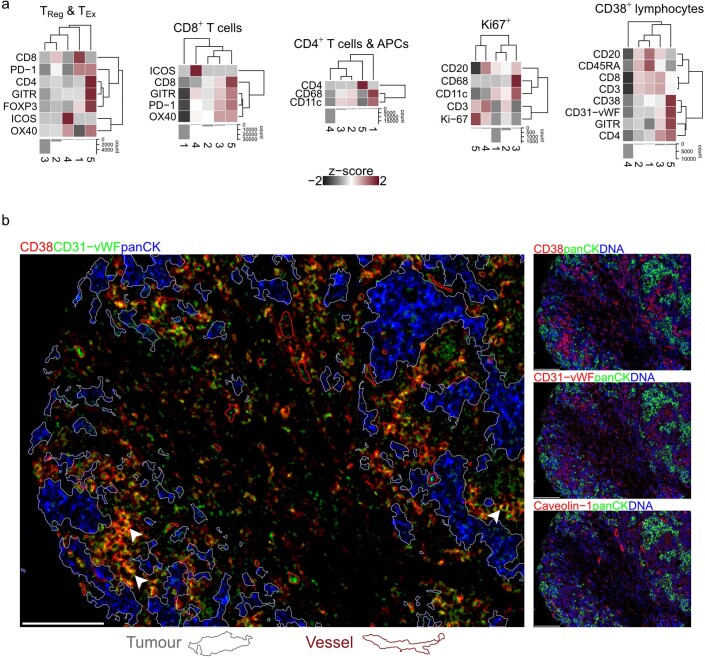
Subclustering of ambiguous cell phenotypes. **a**, Heatmaps of median expression values for five subclusters within each cell phenotype (z-score scaled within each cell phenotype; clipped at -2 and 2). **b**, Example of CD38^+^ cells that also express CD31-vWF (yellow pixels; white arrows). Most of these cells are not adjacent to blood vessels (outlined in red) indicating that coexpression of CD38 and CD31-vWF is not explained by proximity to endothelial cells. Epithelial (tumor) mask outlined in white. Scale bars represent 100 µm.

Major TME cell types (lymphoid, myeloid and stromal) were also subclassified. Lymphoid subclassifications were helper T cells (CD4^+^), cytotoxic T cells (CD8^+^), T_reg_ cells (FOXP3^+^), T cells expressing checkpoint proteins (including PD-1), B cells and CD38^+^ lymphocytes. Myeloid cells included macrophages, granulocytes and other CD11c^+^ antigen-presenting cells (APCs) such as dendritic cells. Consistent with past work^26^, there was notable stromal cell heterogeneity based on the expression of SMA (distinguishing myofibroblasts), FSP1 and PDPN (distinguishing fibroblast subsets). We further characterized T cells, CD38^+^ cells and Ki-67^+^ cells by clustering each into five subclusters (Extended Data Fig. 3a). Subclustering revealed that T cells were characterized by heterogenous checkpoint expression profiles and that T_reg_ cells were relatively scarce. Proliferating Ki-67^+^ TME cells were a combination of lymphoid and myeloid subsets. CD38^+^ cells coexpressed CD31-vWF (detected by a mix of two antibodies, one targeting CD31 and another Von Willebrand factor (vWF)), and subclustering confirmed that a small subset showed high levels of expression of both CD38 and CD31-vWF (Fig. 2c). To determine whether this pattern of coexpression was due to CD38^+^ cells overlapping with endothelial cells (which are CD31-vWF^+^), we inspected images in which they were abundant. In addition to endothelial cells, we found CD31-vWF expression among infiltrating leukocytes, which were also positive for CD38^+^ (Extended Data Fig. 3b). Coexpression on leukocytes is consistent with previous characterization of reactive plasma cells^27^. These analyses show that coexpression of CD38 and CD31-vWF was not due to overlap with endothelial cells.

**Extended Data Fig. 4:**
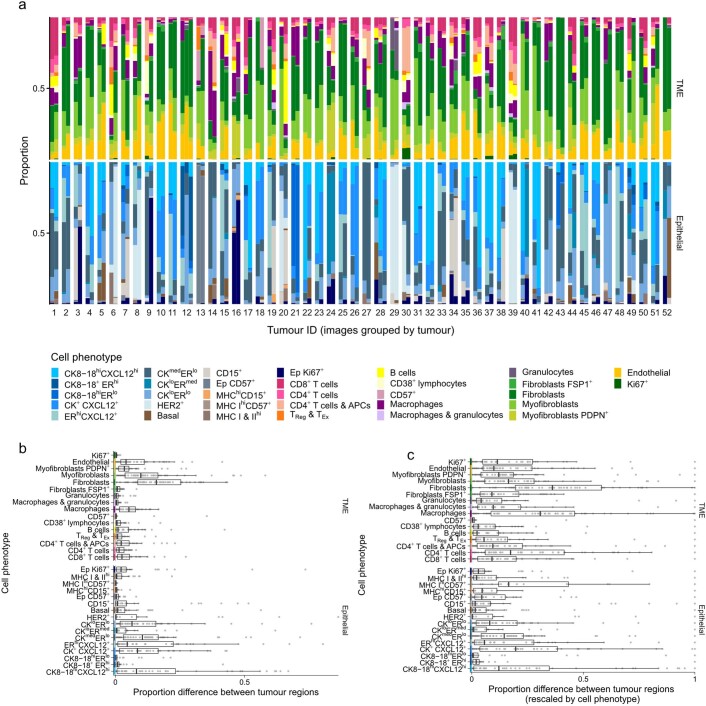
Comparison of cell phenotypic composition among 52 tumors represented by more than one sample. **a**, Stacked bar plots of cell phenotypic composition (separately for epithelial and TME cells). Columns are paired by tumor for comparison. Tumor order (x axis) is arbitrary. **b**, Box plots of proportion differences (as positive values) computed separately for epithelial and TME cells (*n* = 52 tumors). **c**, Box plots of proportion difference rescaled to between zero and one within each cell phenotype (*n* = 52 tumors). For box plots, boxes are 25th, 50^th^, and 75th centiles; whiskers are 75th centile plus 1.5x interquartile range and 25th centile less 1.5x interquartile range. Data points beyond whiskers are outliers.

Our data allowed us to explore spatial intratumor heterogeneity. Although most tumors were represented by a single TMA spot, 52 of the 693 tumors were represented by at least two spots (two tumors were represented by three; Extended Data Fig. 4). Tumor composition was generally well conserved between spots, but there were exceptions where an abundant cell phenotype was absent from one region (e.g., tumors 28 and 52). TME composition was better conserved across regions than were epithelial cell phenotypes. Sampling error, however, is a limitation of our study.

Taking population diversity as an indicator of functional complexity, we investigated how cellular phenotypic diversity differed between tumor subtypes. We used the Shannon diversity index^28^ as a global cell diversity metric and compared diversities of epithelial and TME cells separately using linear models. First, we analyzed tumor and adjacent normal tissue and found that epithelial diversity was lower in normal regions than in tumors but that there was no significant difference when comparing TME cells in tumor versus normal breast tissue (Fig. 2d). This finding indicates a greater deviation from normal for epithelial cells as compared to TME cells. This may be explained by a substantial role for tissue resident cells in populating the TME, where the expansion or contraction of subpopulations occurs in sync to maintain overall cell diversity.

Molecular subtypes of breast cancer reflect the intertumoral heterogeneity of the disease and are also known to be characterized by distinct tumor ecosystems^11^. Therefore, we investigated whether ecosystem diversity of breast cancer subtypes differed in terms of the contribution of epithelial or TME cells both in subtypes based on tumor transcriptomes that closely map to clinical subtypes defined by ER and HER2^29^ and in integrative clusters defined by driver copy-number alterations^21^. After adjustment for multiple testing, there was little difference in epithelial cell diversity among breast cancer subtypes (Fig. 2d). By contrast, TME cell diversity differed markedly; there was higher diversity among ER-negative subtypes (basal; IntClusts 4, 5 and 10) and lesser diversity among indolent estrogen receptor (ER)-positive subtypes (luminal A; IntClusts 3 and 8). These findings show that breast tumors are characterized by TMEs that differ in the compositional complexity of their cell populations.

### Tissue interfaces mark spatial phenotypic transitions

**Fig. 3:**
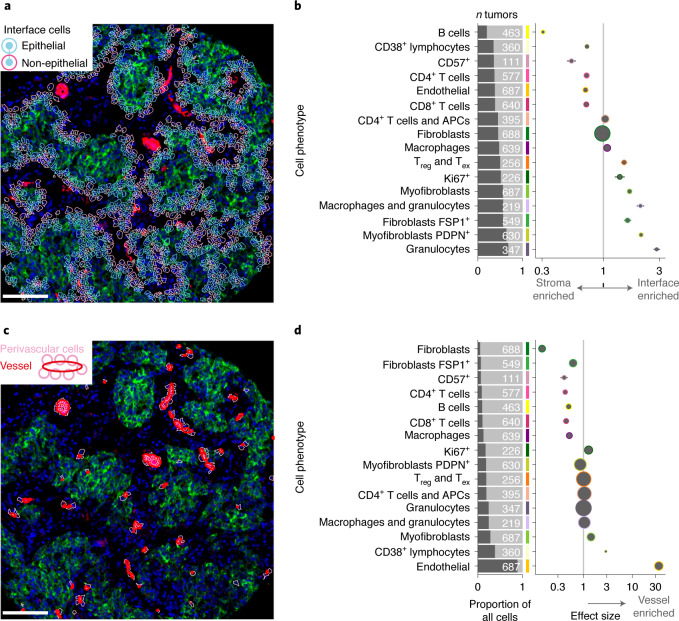
Cell phenotypic transitions at tissue interfaces. **a**, Example image illustrating identification of tumor–stroma interface. Scale bar, 100 µm. **b**, Estimates from generalized linear models of cell types enriched at the tumor–stroma interface. Horizontal lines are 95% confidence intervals, and circles are point estimates. Circles with a colored outline indicate estimates associated with an adjusted *P* < 0.05; from two-sided tests for the linear model regression term; *P* values were adjusted for multiple testing using the Benjamini–Hochberg method. Circle size is inversely proportional to the standard error. Analyses were limited to tumors that contained the cell phenotype of interest; the number of tumors included in each model is depicted in the adjacent bar chart. **c**, Example image illustrating the perivascular interface. Scale bar, 100 µm. **d**, Same as **b** but for cell types enriched in the perivascular space. Horizontal lines are 95% confidence intervals, and circles are point estimates.

**Extended Data Fig. 5:**
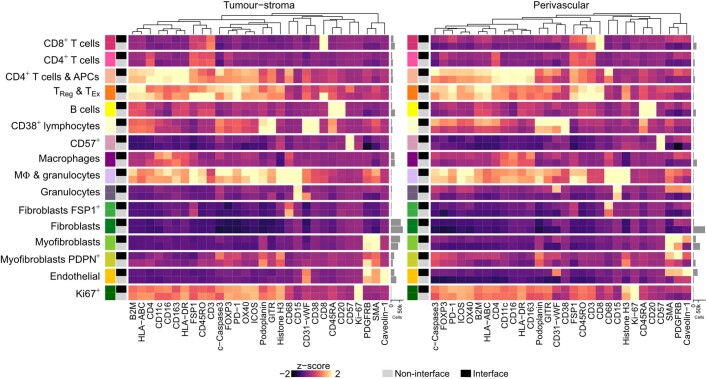
Comparison of expression profiles by cell presence or absence from tissue interfaces. Heatmaps of cell phenotype expression profiles separately for cells present at a tissue interface and those absent from an interface (columns are z-score scaled and clipped at -2 and 2).

Solid tumors organize into compartments (tumor, stroma and vasculature). Cells at interfaces between compartments may participate in reciprocal signaling, and this may alter their phenotypes. Cells may also migrate to an interface because of a secreted factor. Both mechanisms affect TME structure. To investigate how compositional complexity was manifested in space, we categorized cells as either in contact with an interface or not and compared cell compositions of the resulting two categories using generalized linear models. All TME cell phenotypes were significantly enriched or depleted at the tumor–stroma interface (Fig. 3a,b), but this varied dramatically in degree. To determine whether any enriched phenotype was driven by altered expression due to poor cell segmentation encompassing portions of adjacent epithelial cells, we compared cell phenotype expression profiles by whether cells were located at the interface or not (Extended Data Fig. 5). No systematic differences supported this possibility. Myofibroblasts, including those that expressed PDPN, were significantly enriched at the interface, whereas fibroblasts and endothelial cells were depleted (Fig. 3b). Some lymphoid cell phenotypes (CD4^+^ T cells, CD8^+^ T cells, CD38^+^ lymphocytes and B cells) were also depleted at the interface, with B cells showing the greatest interface depletion of all phenotypes (Fig. 3b). This picture of peritumoral stromal activation and lymphocytic depletion supports a model of lymphocytic exclusion mediated by myofibroblasts^30^, consistent with reports of contractile matrix-producing fibroblasts^31^.

We also investigated whether perivascular cells differ from other cells in the TME. Circulating leukocytes infiltrate the TME by traversing the perivascular space. Soluble factors in the peripheral blood and draining lymph fluid may also influence the perivascular cell population. We identified perivascular cells as those in direct contact with a vessel (defined by a pixel mask) and compared their composition to other TME cells (Fig. 3c). As expected, perivascular cells were massively enriched for endothelial cells (Fig. 3d). Because the quantification of endothelial cells and vessels was conducted using independent methods (single-cell phenotyping for endothelial cells and pixel classification for vessels), this finding corroborated our cell phenotyping schema. Other than endothelial cells, the most enriched perivascular cell phenotype was CD38^+^ lymphocytes. This finding may be related to the role CD38 plays in mediating the adhesion of lymphocytes to endothelial cells. The cognate ligand of CD38 is CD31 (ref. ^32^), which is expressed by endothelium. Although it is possible that CD38^+^ lymphocytes were caught during their migration into tissue parenchyma from the peripheral blood (diapedesis) when tissues were fixed, our finding that some non-endothelial CD38^+^ cells can also express CD31-vWF (Extended Data Fig. 3b) suggests this finding is best interpreted cautiously, as some CD31-vWF^+^ pixels belonging to leukocytes may have been mislabeled as vascular by our classifier. There was slight enrichment of myofibroblasts in the perivascular space. As at the tumor–stroma interface, CD4^+^ and CD8^+^ T cells showed perivascular depletion, as did macrophages. Among stromal cells, there was depletion of PDPN^+^ myofibroblasts, FSP1^+^ fibroblasts, and fibroblasts (Fig. 3d). Based on these findings, phenotypic transitions associated with the perivascular space may be explained by a combination of the ingress of circulating leukocytes into the tissue and adjacent stromal activation. Therefore, both tumor–stroma and vascular interfaces impact TME structure. Patterns of cell enrichment and depletion indicate that mechanisms operative at these sites explain these differences and indicate that distinct functions are spatially segregated within the TME.

### Systematic discovery of multicellular TME structures

**Fig. 4:**
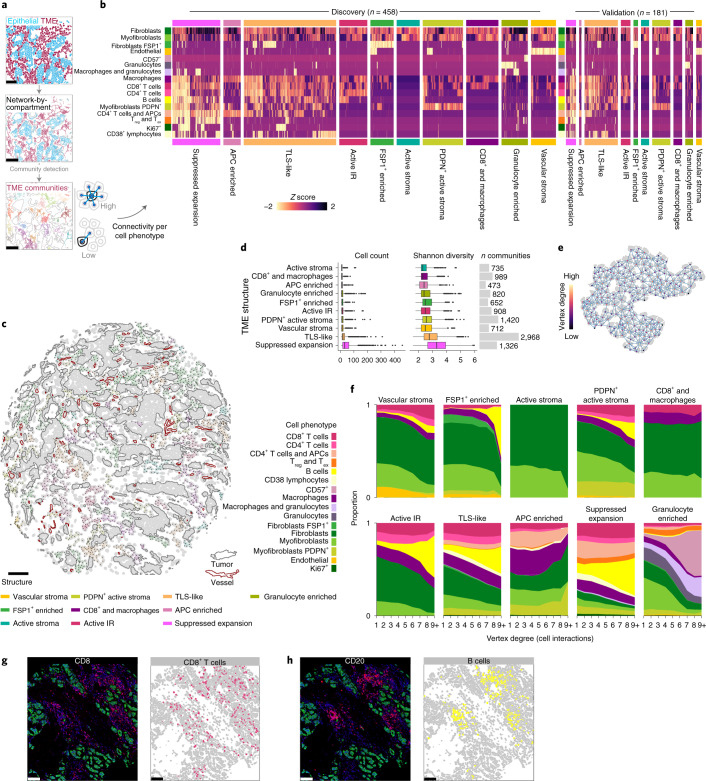
Mapping the landscape of recurrent multicellular structures in the breast TME. **a**, Schematic illustration of community detection of spatial cell networks to identify discrete structures. **b**, Connectivity profiles of ten recurrent TME structures as heatmaps ordered by hierarchical clustering within each structure for the discovery and validation datasets. **c**, Example of TME structures mapped to annotated tissue schematic. Scale bar, 100 µm. **d**, Comparison of number of cells and cell diversity across TME structures for *n* = 616 tumors as box plots (boxes show 25th, 50th and 75th centiles; whiskers indicate 75th centile plus 1.5× interquartile range and 25th centile less 1.5× interquartile range; data beyond whiskers are outliers). **e**, Schematic illustration of the principle of vertex degree (number of incident edges or interactions per cell). **f**, Stacked area plots of cell compositions across different categories of vertex degree per TME structure. **g**,**h**, Example images and schematics of cell phenotype illustrating B cell aggregation compared to diffuse T cell distributions. Scale bars, 100 µm.

**Extended Data Fig. 6:**
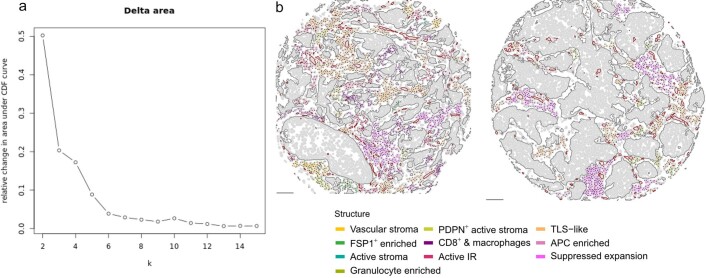
Consensus clustering and examples of tumor microenvironment structures. **a**, Scatter plot of the change in cumulative density function (CDF) between clustering solutions (‘k’ on the x axis). A decrease in CDF indicates an increase in clustering consensus. **b**, TME structures depicted as colored spatial graphs on schematic maps of two METABRIC tumors. Scale bars represent 100 µm.

The TME is a dynamic ecosystem where diverse cells self-organize in response to short and long-range signals to perform specific functions. To identify TME structures while accounting for higher-order cell interactions (spatial relationships) and functional properties (cell phenotype), we represented all cell–cell contacts as a network with cells as vertices and cell–cell contacts as edges^12,33^. Using community-detection methods, we identified highly connected subgraphs that represented the spatial relationships of discretized TME structures. To account for phenotypic differences, the number of cell–cell contacts (connectivity) of each cell phenotype was computed for each subgraph. The resulting profiles of connectivity were then used to classify subgraphs in a subset of 458 tumors from one of the two contributing centers using hierarchical clustering (Fig. 4a). We used a consensus clustering approach to establish a statistically robust number of clusters^34^ and found that ten groups reasonably represented variability in connectivity profiles (Fig. 4b,c and Extended Data Fig. 6). Phenotypic features that distinguished these structures were related to the nature of the stroma and leukocyte composition.

Several structures were characterized by a scarcity of leukocytes but variable stromal activation and vascularization: ‘FSP1^+^ enriched’, ‘active stroma’, ‘PDPN^+^ active stroma^’^, and ‘vascular stroma’. There were several variants of structures indicative of an active IR, defined here as the presence of both cytotoxic and T helper cells. These structures differed by depletion or enrichment of specific cells and included ‘active IR’, ‘CD8^+^ and macrophages’, ‘granulocyte enriched’, and ‘APC enriched’, or had complex heterocellular connectivity profiles reminiscent of TLSs (‘TLS-like’). Another structure with a complex heterocellular profile showed enrichment for T_reg_ cells, cells that expressed immune checkpoint proteins such as PD-1, and proliferating cells and is referred to as the ‘suppressed expansion’ structure. This structure may represent a niche for dysfunctional T cells, which are characterized by expression of immune checkpoints and capacity for replication^35^.

To validate these structures in independent data, we used a random forest classifier (trained on discovery data) to label TME subgraphs from 181 tumors from the second contributing center (Fig. 4b). All ten structures were identified in comparable proportions with similar connectivity profiles. Comparisons of the distributions of cell number and population diversities between TME structures (Fig. 4c) showed that suppressed expansion and TLS-like structures were notably larger on average than other structures, with some exceeding 200 cells. Similarly, their cell population diversity was the highest among all structures (Fig. 4d).

**Extended Data Fig. 7:**
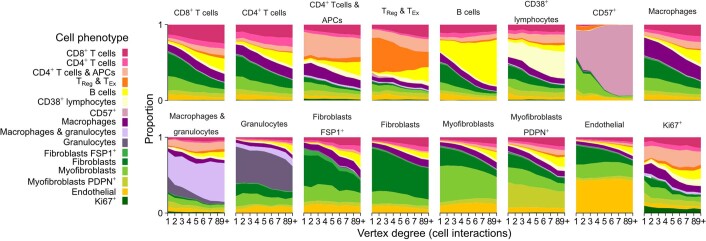
Cell-centric trends of phenotypic composition by number of cell interactions (vertex degree). Stacked area plots for each TME cell phenotype depicting trends of phenotypic composition by the number of cells contacting the cell phenotype of interest.

We investigated whether TME structures differed in terms of the spatial arrangement of different cell phenotypes. We asked, for example, whether certain cell phenotypes were typically peripheral or central. To investigate differences in spatial arrangements while accounting for the complex asymmetries of TME structures, we categorized cells according to their numbers of contacts (vertex degree) and compared the phenotypic compositions of each category (Fig. 4d). Two patterns of intercellular organization emerged: one where cell composition remained relatively constant and another where one cell phenotype showed dramatic expansion (Fig. 4f). Most notably, B cells occupied a greater share of overall cell composition as the number of contacting cells increased. This effect was apparent, to varying degree, across five TME structures: ‘FSP1^+^ enriched’, ‘PDPN^+^ active stroma’, active IR, suppressed expansion and TLS-like. In contrast, T cells occupied a relatively stable share of cell composition across substructures. We repeated this analysis from a cell-centric rather than TME-centric perspective and confirmed both trends (Extended Data Fig. 7). These findings indicate that B cells aggregate in tumor tissues, whereas T cells are distributed more diffusely, and that the size of this effect differs between TME structures (Fig. 4f–h).

### Genomic features are associated with TME structures

Genomic alterations within tumor cells may induce changes in the TME, or, conversely, features of the TME may select for alterations by changing the landscape of cancer cell fitness. To investigate this reciprocal relationship, we determined whether molecular breast cancer subtypes and somatic genomic alterations (mutations and copy-number alterations) were associated with TME structure. We used two complementary approaches: we identified those genomic features most enriched among TME structures, and we investigated the ability of both epithelial and TME features to predict genomic alterations.

**Extended Data Fig. 8:**
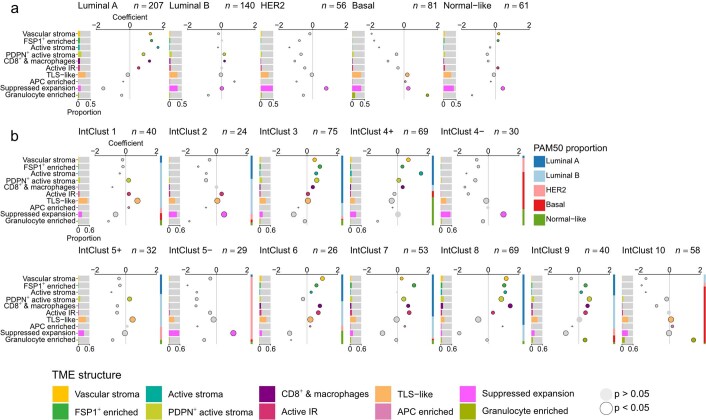
Associations between TME structures and molecular breast cancer subtypes. **a**, Bar charts of distributions of TME connectivity by TME structure for breast cancer intrinsic subtypes (*n* = 545 tumors). Bubble plots represent coefficients from linear models. Size is inversely proportional to the precision of the estimate; colored circles are significantly enriched TME structures; black outlines denote an adjusted p-value of < 0.05 for that term; p-values are for two-sided tests and were adjusted for multiple testing using the Benjamini–Hochberg method; horizontal lines are 95% confidence intervals. **b**, Bar charts of distributions of TME connectivity by TME structure by IntClust subtypes (*n* = 545 tumors). Bubble plots represent coefficients from linear models. Size is inversely proportional to the precision of the estimate; colored circles are significantly enriched TME structures; black outlines denote an adjusted p-value of < 0.05 for that term; p-values are for two-sided tests and were adjusted for multiple testing using the Benjamini–Hochberg method; horizontal lines are 95% confidence intervals. For reference, the stacked bar to the right of each plot illustrates the composition of tumors within that IntClust subtype by PAM50 subtypes.

We first asked whether TME structures differ between breast cancer subtypes (Extended Data Fig. 8). Patterns of enrichment were highly distinctive between subtypes, with ER status exerting greatest influence on TME structure. Because most dysfunctional T cells and T_reg_ cells resided in suppressed expansion structures, we characterized their enrichment among tumor subtypes. Among intrinsic breast cancer subtypes based on transcriptomic groups^29^, mapping closely to clinical subtypes defined by ER and HER2, suppressed expansion structures showed variable enrichment in all groups except luminal A tumors, among which they were significantly depleted. Analysis of IntClust subtypes defined by driver copy-number aberrations^21^ revealed that enrichment for suppressed expansion structures was most marked in ER-negative tumors irrespective of HER2 status (IntClust 4− and 5−) and in IntClust 2 tumors (an aggressive ER-positive subgroup driven by 11q13/14 amplification^21^).

**Fig. 5:**
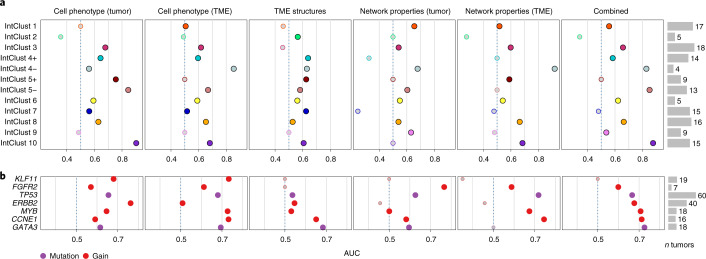
Genomic breast cancer subtypes and driver somatic alterations are associated with TME structures. **a**,**b**, Scatter plots of AUC receiver-operating characteristic statistics for performance of different categories of predictors in classifying genomic breast cancer subtypes in an out-of-sample dataset. **b**, Same as **a** but with tumor subtypes substituted for driver somatic alterations. Depicted are predictors with AUCs of >0.7 for at least one model.

We reasoned that comparing the performance of different categories of predictors would enable comparison of the relative contribution of TME features to subtype designation. For samples with available molecular subtyping data, we used regularized logistic regression to fit separate models for each IntClust subtype, using the data from one of the two METABRIC centers for training (*n* = 390 patients), data from the other for testing (*n* = 147 patients) and computed area-under-the-curve (AUC) receiver-operating characteristic statistics using the test data to compare performance. Categories of predictors, which were cell phenotypes, TME structures and network properties (summary statistics describing spatial features of the subgraphs within a tumor), were computed separately for tumor and TME cells (Fig. 5a). Differential predictive accuracy was most marked for IntClust 4− (ER-negative tumors with few copy-number aberrations), which were better predicted by TME cell phenotype than by tumor cell phenotype (AUC < 0.6 v > 0.8) but were best predicted by the network properties of TME subgraphs (AUC 0.91), highlighting the importance of TME spatial features in defining the biology of this subtype.

**Extended Data Fig. 9:**
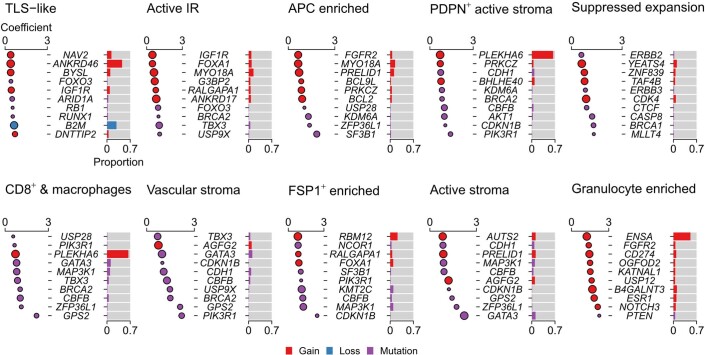
Associations between TME structures and driver somatic alterations. Top ten most enriched somatic alterations per TME structure (*n* = 545 tumors for copy-number aberrations and *n* = 530 tumors for mutations). Bubble plots where circles depict generalized linear model coefficients. Size is inversely proportional to the precision of the estimate. Black outlines denote an adjusted p-value of < 0.05; p-values are for two-sided tests and were adjusted for multiple testing using the Benjamini–Hochberg method. Horizontal lines (where visible) indicate 95% confidence intervals. Bar charts illustrate the proportion of analyzed samples that harbor the alteration.

We also tested for associations with driver somatic alterations. Although driver alterations are associated with tumor subtype^36^, their co-occurrence is not exact. Hence, we analyzed these features independent of tumor subtype. Among somatic alterations most enriched for the suppressed expansion structure were *BRCA1* and *CASP8* mutations (Extended Data Fig. 9). These may represent both sides of the cancer cell–TME dynamic. Mutations of *BRCA1* impair homologous recombination-mediated double-strand break repair and are associated with distinctive genomic profiles involving specific mutational signatures, large deletions and indels^37^. Some of these features may lead to a more vigorous adaptive IR^38,39^. In contrast, mutations of *CASP8* protect against apoptosis induced by engagement of Fas receptor by its cognate ligand expressed on cytotoxic T cells, effectively representing an immune escape lesion^15^. *BRCA2* mutations were also associated with four other TME structures: active IR, ‘CD8^+^ and macrophages’, ‘vascular stroma’ and ‘PDPN^+^ active stroma’. This further supports a link between compromised DNA damage repair and TME modulation. Gains of *CD274* (encoding PD-L1) were among the top ten hits for granulocyte enriched, and TLS-like was associated with loss of *B2M* (encoding β2-microglobulin, a component of MHC-I), corroborating past work^11,15,19^ and indicating that these alterations aid immune escape. Notably, mutations of *CDH1* were associated with distinct stromal features: ‘PDPN^+^ active stroma’ and ‘vascular stroma’. Mutations of *CDH1* (encoding E-cadherin) are characteristic of lobular breast cancer that shows single-file cancer cell growth. This observation suggests that the TME structure of lobular breast cancer is distinctive due to variable stroma and heterocellular leukocytic infiltrates with distinct spatial organization.

To further investigate the genotype–tissue phenotype relationship, we asked whether various tissue features were impacted disproportionately by particular somatic alterations. Our hypothesis was that some alterations influence cell phenotype, whereas others influence patterns of growth and spatial relationships, and this may differ between epithelial cells and those of the TME. To evaluate the relationship with somatic alterations, we used a series of regularized regression models to predict alterations based on different categories of tissue features (cell phenotype, structure, network properties) separately for tumor cells and TME cells (Fig. 5b). As expected, we observed high prediction accuracy for *ERBB2* gains but only when tumor cell phenotypes were used as predictors (AUC 0.76). Mutations of *TP53* were better predicted by TME network properties (mainly spatial characteristics; AUC 0.72) than tumor cell phenotype (AUC 0.65), indicating that there are TME spatial features that are highly characteristic of *TP53*-mutant tumors. Gains of *CCNE1* were also predicted with comparable accuracy by TME network properties (AUC 0.74), linking this alteration to distinct TME spatial orientation. Together, our findings reveal that there are complex relationships between somatic alterations and the TME suggestive of an ongoing dynamic due to differential cancer cell fitness in the context of specific TME structures.

### TME structure is predictive of clinical outcome

**Fig. 6:**
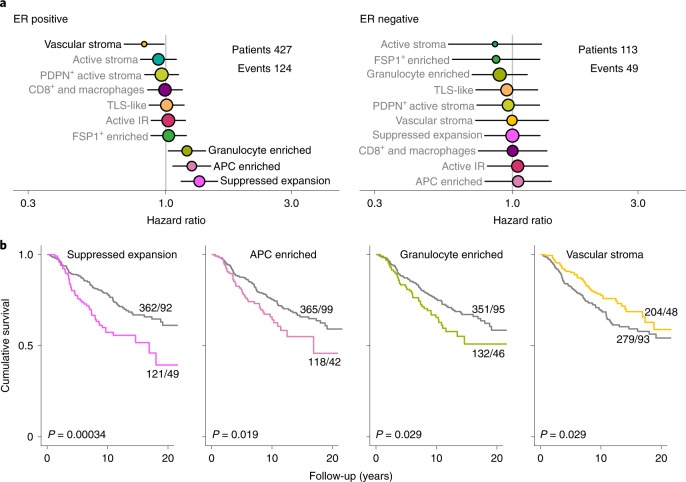
Prognostic impact of TME structures. **a**, Hazard ratios for all TME structures separately by estrogen receptor status (427 patients with ER-positive disease and 113 with ER-negative disease) and adjusted for HER2 status. Circles represent point estimates of hazard ratios, circle size is inversely proportional to the standard error and horizontal lines depict 95% confidence intervals. The grey and black text distinguishes between statistically significant and non-significant associations. **b**, Survival plots for TME structures where tumors were classified according to whether the structure was present or absent. All depicted *P* values are from log-rank tests for *n* = 483 patients.

Finally, we investigated whether TME structures are indicative of clinical outcome. We estimated hazard ratios for disease-specific survival (adjusted for HER2 status) associated with each TME structure (Fig. 6a,b). Past work has shown that the prognostic impact of infiltrating leukocytes significantly differs in ER-positive versus ER-negative breast cancer^40^; hence, we conducted separate analyses by ER status. Four TME structures were significantly associated with outcome in ER-positive disease. In contrast, no structures were significantly associated with outcome in ER-negative disease. The lack of detected associations in ER-negative disease, however, is probably due to lesser statistical power (124 versus 49 events). Of the four structures associated with outcome in ER-positive tumors, three were associated with poor prognosis (granulocyte enriched, APC enriched and suppressed expansion), and one was associated with favorable outcome (‘vascular stroma’). The prognostic factor associated with the worst prognosis was suppressed expansion; this is the TME structure in which most T_reg_ cells and dysfunctional T cells were located. The suppressive function of T_reg_ cells may explain this association, as their abundance has been linked to poor outcome^41^. It is also possible that the presence of large aggregates of dysfunctional T cells are indicative of tumor cells that can endure ongoing immune attack and cause chronic stimulation of cytotoxic T cells. Likewise, the poor prognostic impact of the APC-enriched structure could be related to the abundance of macrophages, which are known to orchestrate immunosuppressive effects^42^ and have previously been linked to poor outcome in breast cancer^41^. Past work also suggests that there may be a nonlinear association between TME features and outcome in ER-positive breast cancer^41^. Although our study is among the largest highly multiplexed imaging studies of cancer tissues, it should be noted that our analyses are insufficiently powered to investigate such effects. Our study does show that the TME is a key determinant of clinical outcome and that distinct facets of TME structure provide complementary prognostic value. Analyses of multiparametric representations of TME structure are likely to increase predictive accuracy in the clinical setting.

## Discussion

High-dimensional tissue imaging of breast tumor tissue revealed that specialized cells of the TME organize to form varied structures that recur across tumors. Tissue interfaces and cancer cell-intrinsic factors impact formation of these structures, and the structures in turn exert selective pressure on cancer cells. Given the intimate link between structure and function, it follows that these multicellular structures offer a window on the functional state of the TME, and their associations with outcome suggest their characterization could be useful for patient stratification.

Dysfunctional T cells are characterized by high expression of immune checkpoint factors, a gradation of transitional states and proliferative capacity^35,43^. This population may be spatially segregated alongside other diverse leukocytes, including APCs, in suppressed expansion structures. Spatial segregation also occurs within the renal cell carcinoma TME, where progenitor-like T cells preferentially exist and replicate in niches populated by APCs^44^. Together with our finding in breast cancer, this suggests that groups of dysfunctional T cells congregate in space and that diverse leukocytes contribute to a local microenvironment that regulates function and replication state of these T cells. Recent findings also raise the possibility that cells within this population are more likely than other cells of the TME to harbor T cell receptors specific to tumor cell-related epitopes^45^. Suppressed expansion structures may therefore contain T cells that could be targeted by immunotherapy.

The repertoire of TME structures differed between IntClust tumor subtypes, corroborating our past findings^11,46^. IntClust subtypes are based on driver copy-number aberrations^21^. Divergent TME structures are therefore indicative of crosstalk between tumor cells and cells of the microenvironment, and this crosstalk impacts intercellular organization. We also uncovered associations with somatic alterations that suggest a reciprocal dynamic sculpts the TME and tumor cells in tandem. Mutations of *BRCA1* and *CASP8* were among the top ten most enriched in tumors with suppressed expansion structures. Cancer cells with compromised DNA damage response due to a *BRCA1* mutation show distinctive genomic alterations^37^ that may result in the display of neoantigens leading to targeting by adaptive immune cells. This could favor cancer cells with defective *CASP8*, as *CASP8*-mutant cells are resistant to extrinsic apoptosis induced by cytotoxic T cells^47^. Past pan-cancer analyses also show that cells with *CASP8* mutations escape immune surveillance^15^. Co-occurrence of mutations that increase immunogenicity (e.g., *BRCA1*) with those enabling immune escape (e.g., *CASP8*) suggests a model of cancer progression where immune-mediated selection is a later event, in the setting of an otherwise immunogenic population. Future analyses of high-resolution sequencing data with temporal inference of mutations^48^ together with high-dimensional imaging of the TME should enable deeper interrogation of this dynamic and may reveal the extent to which the selection of driver alterations is sensitive to TME context.

The association between TLSs and immunotherapy response^3–5^ suggests that a clinical assay to detect them in situ is needed. TLSs contain a B cell-rich center, but our findings indicate that B cell aggregation is a general feature of the TME rather than a specific feature of TLSs, at least in breast cancer. B cell aggregates were observed in five TME structures (FSP1^+^ enriched, PDPN^+^ active stroma, active IR, suppressed expansion and TLS-like). These structures had opposing patterns of enrichment among tumor subtypes. For example, TLS-like structures were characteristic of basal-like tumors, whereas active IR structures were characteristic of luminal A tumors, which implies that disparate underlying mechanisms lead to B cell aggregation in these structures and corroborates the finding that B cell function is dependent on spatial context^49^. For these reasons, assays for TLSs using in situ B cell counts as a surrogate will lack specificity unless adjusted for wider TME context. This highlights the challenge of designing pragmatic and quantitative assays for complex multicellular structures; standardization of both experimental and computational workflows will be necessary.

To identify new therapeutic targets, it will be critical to understand the dynamic functional states of the TME. In our study, high-dimensional imaging revealed how the specialized cells of the breast TME organize in space, how this organization varies across tumors and how various structures impact clinical outcomes. Our approach enables a deeper understanding of structural immunity in tumors^50^ and may help identify patients likely to respond to therapies that function by perturbing spatial organization of the TME.

## Methods

### Study design, TMA production and metadata

Formalin-fixed paraffin-embedded treatment-naive primary breast tumor tissue from patients recruited to the METABRIC study^21^ was used for this work. All samples were obtained with written, informed patient consent, and the study protocol was approved by the NRES Cambridgeshire 2 Research Ethics Committee (REC ref. 07/H0308/161). The study was an observational case series, and tumor samples were all excised before systemic therapy. Sample size was determined by whether suitable formalin-fixed paraffin-embedded tissue was available for research; women with breast cancer (mean age, 62 years; range, 22–96 years) were included and were not compensated for participation; there were no exclusion criteria. To facilitate throughput and minimize experimental batch effects, tumor tissues were represented in TMAs. Briefly, suitable areas of invasive cancer were selected by a pathologist (E.P.) using whole hematoxylin and eosin-stained slides. These areas were punched using a manual microarrayer and inserted into a receiver TMA block. Most tissue spots (93%) were 0.6 mm in diameter, but one TMA block contained spots that were 1 mm in diameter (7%). A total of 794 tissue spots from 718 patients were analyzed. Targeted sampling and manual inspection of images by a pathologist (H.R.A.) showed that, of these, 31 spots only contained histologically normal breast tissue and 14 only contained in situ carcinoma. This left a total of 749 images corresponding to 693 patients (635 tumors were represented by one tissue spot, 55 by two and 3 by three), of which 639 contained epithelial cells. Patients were treated at two participating UK centers (contributing 500 and 193 patients, respectively). A subset of samples were associated with previously generated genomic data available in the public domain, including gene expression microarrays (Illumina bead arrays; 587 patients), high-resolution array comparative genomic hybridization (587 patients) and targeted sequencing of breast cancer genes (568 patients) data. Detailed protocols for these genomic assays are available in their corresponding publications^21,36^. Recently updated clinical data, also in the public domain, were linked to analyzed samples^22^.

### Antibody panel design and metal conjugation

Antibody panel design (including the description of antibodies, concentration, clone information and metal isotype tag used) are provided in Supplementary Table 1. All antibody–metal conjugations were conducted with the Maxpar labeling kit (Fluidigm). Antibody concentration was titrated (100–500 µg ml^−1^) using a Nanodrop (Thermo Scientific), and conjugated antibodies were stored in a Candor antibody stabilizer (Candor Bioscience) at 4 °C (ref. ^11^). Antibody staining patterns and concentration were evaluated by inspection of IMC images from a variety of tissues, including tonsil, normal breast and breast cancer.

### Tissue labeling and IMC

The procedures for tissue staining and analysis of antibody-labeled sections by imaging mass cytometry were as follows: 4 µm tissue sections were dewaxed and rehydrated through an alcohol series and subjected to epitope retrieval using Tris-EDTA buffer (pH 9) at 95 °C using a decloaking chamber for 30 min, tissues were then labeled with metal-tagged antibodies by overnight incubation at 4 °C (except for anti-ER, which was detected by a metal-tagged secondary antibody to boost signal) and iridium DNA intercalator (Fluidigm, 201192B) was used for detection of DNA. Finally, air-dried tissues were ablated using an imaging mass cytometer (Fluidigm).

### Image processing

Ion counts (corresponding to bound antibody abundance) were recorded in TXT files and converted to TIFF image stacks using an established workflow^51^. Taking advantage of the multidimensional nature of the data, we first classified images at the level of pixels. Pixels were manually labeled according to the structure of interest (single cells, tumor regions or vasculature), and unlabeled pixels were classified based on random forest models implemented in Ilastik^52^. For single-cell segmentation, we labeled pixels into three classes: nuclear, cytoplasm and membrane, and background (acellular regions). A tumor region classifier was generated using all measured cytokeratins (pan-CK, CK8-18 and CK5), and a vessel classifier was generated using CD31, SMA and Caveolin-1. In addition, small aggregates of hot pixels corresponding to Ho165 (ER) were identified by a further pixel classifier (Supplementary Table 2). These classifiers were used to generate probability maps as RGB tiff files for export to CellProfiler^53^. For tumor and vessel regions, probability maps were segmented using a manual global thresholding strategy (threshold set at 0.5) and clumped objects separated based on signal intensity. Single-cell segmentation was conducted by first detecting nuclei (primary objects; manual global threshold at 0.5) and then detecting whole cells (secondary objects) by propagation-based expansion of nuclear regions (to encompass the membranocytoplasmic signal and stopping at the cell edge according to signal intensity gradients). Single-cell expression values were defined as the mean ion count encompassed by a whole-cell mask. Expression values for two markers (ER and SMA) were defined by region masks (nucleus and cytoplasm, respectively) rather than whole-cell values to account for non-specific background signal. Cell-to-cell relationships were defined as adjacent cells falling within a distance of 8 µm. Objects (nuclei, whole cells, tumor and vessel regions and hot pixel aggregates) were defined as ‘related’ if any overlapping pixels were identified. Cells affected by hot pixel aggregates according to this definition were removed from further analyses.

### Spillover compensation

Modest channel crosstalk is known to occur in mass cytometry experiments owing to small isotopic impurities in metal stocks. To adjust for this effect, we spotted all metal-conjugated antibodies onto an agarose-coated glass slide and measured isotopic composition by IMC. We used the Bioconductor CATALYST package to generate a ‘spillover matrix’ from these data, which allowed for adjustment of cross-channel spillover in single-cell expression data by a non-negative least-squares regression model using CATALYST^54^.

### Manual curation and single-cell phenotyping

To classify cells into distinct phenotypes, we adopted an automated approach assisted by manual curation. On the basis that the starkest phenotypic separation in breast cancer tissue is whether cells are epithelial or not, we first meticulously segregated cells according to this criterion. To reliably identify epithelial cells across tumors, we used two complementary classification methods. Firstly, we fit a two-component Gaussian mixture model to log-transformed pan-cytokeratin counts per image to distinguish positive from negative cells. Secondly, we identified all cells related to a tumor mask as defined using the approach described above. A pathologist then inspected pairs of annotated images (where epithelial cell outlines were highlighted as classified by one method or the other) and identified which method best classified cells as epithelial using cell morphology and expression of cytokeratins as a guide. During this process and assisted by complete image stack data, tissue spots that only contained histologically normal tissue or in situ carcinoma were also flagged. Having distinguished cells as epithelial or non-epithelial, automated cell clustering was conducted using distinct repertoires of proteins for epithelial and non-epithelial cells (epithelial: Histone H3, CK5, HLA-DR, CK8-18, CD15, HER2 (3B5), Podoplanin, HER2 (D8F12), B2M, ER, CD57, Ki-67, CXCL12, HLA-ABC, pan-CK and c-Caspase3; non-epithelial: Histone H3, SMA, CD38, HLA-DR, CD15, FSP1, CD163, ICOS, OX40, CD68, CD3, Podoplanin, CD11c, PD-1, GITR, CD16, CD45RA, B2M, CD45RO, FOXP3, CD20, CD8, CD57, Ki-67, PDGFRB, Caveolin-1, CD4, CD31-vWF, HLA-ABC and c-Caspase3). Expression values were arc-hyperbolic-sine transformed using 0.8 as a cofactor and clipped at the 99th centile before clustering. We deployed a clustering strategy similar to that previously described^11^. Briefly, self-organizing maps^55^ were used to segregate cells into 1,225 groups. Median expression values of these self-organizing map groups were then used as input to Phenograph^56^ to identify cell phenotypes, which were finally mapped back to single cells. Inspection of heatmaps depicting the *Z*-transformed median expression values for cell lineage markers and IMC images annotated with cell phenotype outlines were used for manual merging of cell clusters. Groups with functionally and morphologically similar characteristics were merged to define cell phenotypes (16 epithelial and 16 TME). Diversity by cell phenotype was computed using the Shannon diversity metric separately for epithelial and non-epithelial cells.

### Comparison of cell phenotype composition at tissue interfaces

To investigate spatial differences in the composition of cells present at tissue interfaces (tumor–stroma and perivascular), cells were first classified as belonging to a given interface or not. We elected to take this approach because it was deemed more robust than a comparison of distances, as measures of distance were severely confounded by limited tissue area. Any non-epithelial cell contacting at least one epithelial (tumor) cell was defined as present at the tumor–stroma interface, whereas perivascular cells were defined as those contacting vessel masks (trained as described above). Generalized linear models were used to compare proportions at tissue interfaces (under a binomal distribution with a logit link function) and weighted by the total cell count to account for the variably precise estimates of cell proportions. Cell proportions were taken as the response variable and whether cells were present at the interface or not as the predictor. *P* values were adjusted for multiple testing using the Benjamini–Hochberg method.

### Identification, classification and characterization of tissue structures

We adopted a systematic approach to identification of multicellular tissue structures. Spatial graphs (networks) were generated separately for epithelial and non-epithelial cells per image. Perivascular cells (those associated with vessel masks) were excluded. Each cell was taken as a vertex (node) and relationships with neighboring cells (identified as described above) taken as edges (links) to encompass all cell relationships within each image. Next, TME graphs (all non-epithelial-to-non-epithelial relationships) were segregated into highly connected communities using a community-detection algorithm based on random walks. To identify similar recurrent structures while accounting for both spatial and phenotypic characteristics, we computed connectivity profiles for each subgraph. A connectivity profile was defined as the number of connections each of the 16 TME cell phenotypes contained in each subgraph. Cell connectivity was chosen over proportion to both account for the arrangement of cells in a structure (the same cell proportion may show high or low connectivity) and avoid normalizing structures by their size. Discovery data (from one center) were grouped using hierarchical clustering by Ward’s method to identify subgraphs with similar profiles. We used consensus clustering^34^ to determine a statistically robust number of TME structures and settled on ten based on the change in cumulative density function across clustering solutions. Descriptive labels were attached to the resulting groups informed by their connectivity profiles. To evaluate the reproducibility of these groups, we trained a random forest classifier on their connectivity profiles and used this to classify subgraphs in the validation dataset (the second center) into the ten groups. To compare spatial features of TME structures, several network property statistics were computed per subgraph, these included: the number of vertices, number of edges, diameter, density, transitivity and assortativity. To compare cell diversity, Shannon diversity by cell phenotype for every subgraph was computed. Intercellular organization within TME structures was investigated by comparison of cell phenotypic composition according to different categories of vertex degree (number of cell–cell contacts), which relied on connectivity to distinguish the contribution of different cells to a subgraph.

### Associations between TME structures and genomic features

To investigate differences in TME structure between breast cancer subtypes, we asked whether different subtype designations could predict the proportion of all TME connections (cell–cell contacts) occupied by each TME structure. To achieve this, we fit generalized linear models for each tumor subtype (under a binomial distribution with a logit link function), where the response variable was the proportion of TME connections and the predictor was tumor subtype (samples were classed as belonging to a subtype of interest or not). Because the total number of cell–cell contacts differed between tumors, the proportions calculated for each TME structure varied in their precision. To account for this variability, models were weighted by the total number of connections per tumor. We also compared the extent to which different derived tissue features (cell phenotype, structure, network properties and a combination of these) could predict IntClust subtypes by training a series of regularized logistic regression models for each IntClust subtype using the data from one of the two contributing METABRIC centers. The performance of these models was assessed by computing receiver-operating AUC statistics for predictions using test data from the other METABRIC center. Similar principles were followed for evaluating associations with somatic driver alterations (mutations, gains and losses). Using existing targeted sequencing data, we selected driver mutations fulfilling previously defined criteria (a ratiometric mutational distribution score of greater than 20)^36^. Only alterations present in at least five sample were included. We identified driver copy-number alterations based on a previously described integrative score^57^ and one oncogene not present in the list (*ZNF703*)^58^. In addition, we included alterations previously implicated as mechanisms of immune escape (gains of *CD274*, loss of *B2M*)^15^ and recurrent deletions (loss of *CDKN2AIP*, *PPP2R2A*, *MTAP*, *PTEN* and *MAP2K4*). A total of 103 alterations were evaluated per TME structure. All alterations were modeled as categorical (present or absent).

### Survival analyses

Associations between tissue structures and disease-specific survival were conducted using Cox proportional-hazards regression models separately by ER status, because ER is known to violate the proportional-hazards assumption, but compliance of other variables was not formally tested. The proportion of connectivity per structure was discretized into four categories; tumors with at least one occurrence of a given structure were separated into tertiles based on the proportion of compartment-specific connections occupied by a structure, whereas tumors lacking a given structure were grouped into a separate baseline category. Models were adjusted for HER2 status (derived from gene expression using a two-component Gaussian mixture model) by including it as a covariate. Log-rank tests were used to assess differences between strata in survival plots where two groups were defined based on whether a given structure was present or not.

### Statistics and reproducibility

Differential abundance of cell phenotype or connectivity proportion by tumor subtype or genomic alteration was tested using generalized linear models (under a binomial distribution with a logit link function), weighted by the total number of observations (either total cells or total cell–cell interactions) per tumor^59^. Survival analyses were conducted using Cox proportional-hazards models, and differences between groups in survival plots were tested using log-rank tests. Where appropriate, adjustment for multiple testing was conducted using the Benjamini–Hochberg method. All statistical analyses were conducted using R version 3.5.1. Representative images of IMC (numeric matrices of isotopic counts) from a single experiment were derived by processing data to TIFF image files, rescaling values between zero and one per image for three markers of interest and representing rescaled values in RGB color space.

### Reporting Summary

Further information on research design is available in the Nature Research Reporting Summary linked to this article.

## Online content

Any methods, additional references, Nature Research reporting summaries, source data, extended data, supplementary information, acknowledgements, peer review information; details of author contributions and competing interests; and statements of data and code availability are available at 10.1038/s41588-022-01041-y.

## Supplementary information

Reporting Summary

Supplementary Table 1 Supplementary Tables 1 and 2.

## Data Availability

Imaging mass cytometry data, derived images and processed single-cell data have been placed in the Zenodo data repository (10.5281/zenodo.5850952). Genomic and clinical data for METABRIC are available at cBioPortal (https://www.cbioportal.org/) or from the respective publications^21,22,36^. METABRIC genomic data are available from the European Genome-phenome Archive under accession numbers EGAS00000000083 and EGAS00001001753.

## Code availability

Image processing and analysis was conducted using software available at https://github.com/BodenmillerGroup/ImcSegmentationPipeline, https://github.com/CellProfiler and https://github.com/ilastik. Cell clustering and spatial network analyses were conducted using the following R packages: FlowSOM (https://github.com/saeyslab/FlowSOM), Phenograph (https://github.com/JinmiaoChenLab/Rphenograph) and igraph (https://github.com/igraph/rigraph). Analysis code is available at Zenodo (10.5281/zenodo.6036188).
