# Supplementary material for: Breast tumor microenvironment structures are associated with genomic features and clinical outcome
Source: Nat Genet. 2022 Apr 18;54(5):660–9. doi: 10.1038/s41588-022-01041-y (PMC7612730; doi:10.1038/s41588-022-01041-y)
Supplement: Supplementary file 1 — Reporting Summary [file 41588_2022_1041_MOESM1_ESM.pdf]

nature research

Corresponding author(s): Bernd Bodenmiller, Carlos Caldas, H. Raza Ali

Last updated by author(s): 14/01/2022

Reporting Summary

Nature Research wishes to improve the reproducibility of the work that we publish. This form provides structure for consistency and transparency in reporting. For further information on Nature Research policies, see our Editorial Policies and the Editorial Policy Checklist.

Statistics

For all statistical analyses, confirm that the following items are present in the figure legend, table legend, main text, or Methods section.

n/a	Confirmed	
☐	☒	The exact sample size (*n*) for each experimental group/condition, given as a discrete number and unit of measurement
☐	☒	A statement on whether measurements were taken from distinct samples or whether the same sample was measured repeatedly
☐	☒	The statistical test(s) used AND whether they are one- or two-sided<br>*Only common tests should be described solely by name; describe more complex techniques in the Methods section.*
☐	☒	A description of all covariates tested
☐	☒	A description of any assumptions or corrections, such as tests of normality and adjustment for multiple comparisons
☐	☒	A full description of the statistical parameters including central tendency (e.g. means) or other basic estimates (e.g. regression coefficient) AND variation (e.g. standard deviation) or associated estimates of uncertainty (e.g. confidence intervals)
☐	☒	For null hypothesis testing, the test statistic (e.g. *F*, *t*, *r*) with confidence intervals, effect sizes, degrees of freedom and *P* value noted<br>*Give P values as exact values whenever suitable.*
☒	☐	For Bayesian analysis, information on the choice of priors and Markov chain Monte Carlo settings
☒	☐	For hierarchical and complex designs, identification of the appropriate level for tests and full reporting of outcomes
☐	☒	Estimates of effect sizes (e.g. Cohen's *d*, Pearson's *r*), indicating how they were calculated

Our web collection on statistics for biologists contains articles on many of the points above.

Software and code

Policy information about availability of computer code

Data collection	The commerically available 'CyTOF software' program (Fluidigm Inc) was used for data acquisition by an imaging mass cytometer.
Data analysis	Image processing and analysis was conducted using software available at https://github.com/BodenmillerGroup/ImcSegmentationPipeline, CellProfiler version 3.1 (https://github.com/CellProfiler), and Ilastik version 1.3.0 (https://github.com/ilastik). Statistical analyses were conducted using R version 3.5.1. Cell clustering and spatial network analyses were conducted using the following R packages: FlowSOM (https://github.com/saeyslab/FlowSOM); Phenograph (https://github.com/JinmiaoChenLab/Rphenograph); igraph (https://github.com/igraph/rigraph). Analysis code is available at Zenodo (https://doi.org/10.5281/zenodo.6036188)

For manuscripts utilizing custom algorithms or software that are central to the research but not yet described in published literature, software must be made available to editors and reviewers. We strongly encourage code deposition in a community repository (e.g. GitHub). See the Nature Research guidelines for submitting code & software for further information.

Data

Policy information about availability of data

All manuscripts must include a data availability statement. This statement should provide the following information, where applicable:

- Accession codes, unique identifiers, or web links for publicly available datasets
- A list of figures that have associated raw data
- A description of any restrictions on data availability

Raw and derived imaging mass cytometry data are available at the Zenodo repository (https://doi.org/10.5281/zenodo.5850952)

Field-specific reporting

Please select the one below that is the best fit for your research. If you are not sure, read the appropriate sections before making your selection.

☒ Life sciences ☐ Behavioural & social sciences ☐ Ecological, evolutionary & environmental sciences

For a reference copy of the document with all sections, see nature.com/documents/nr-reporting-summary-flat.pdf

Life sciences study design

All studies must disclose on these points even when the disclosure is negative.

Sample size	Limited by the availability of suitable sample material.
Data exclusions	None
Replication	Data were generated from two contributing centres and, where relevant, these were divided into training and test data. The primary data generation experiment (imaging mass cytometry of primary breast tumours) was conducted once.
Randomization	There was no randomisation as this was an observational study and no intervention was compared between groups.
Blinding	The study was not blinded because the efficacy of an intervention was not evaluated.

Reporting for specific materials, systems and methods

We require information from authors about some types of materials, experimental systems and methods used in many studies. Here, indicate whether each material, system or method listed is relevant to your study. If you are not sure if a list item applies to your research, read the appropriate section before selecting a response.

Materials & experimental systems

n/a	Involved in the study	
☐	☒	Antibodies
☒	☐	Eukaryotic cell lines
☒	☐	Palaeontology and archaeology
☒	☐	Animals and other organisms
☐	☒	Human research participants
☒	☐	Clinical data
☒	☐	Dual use research of concern

Methods

n/a	Involved in the study	
☒	☐	ChIP-seq
☒	☐	Flow cytometry
☒	☐	MRI-based neuroimaging

Antibodies

Antibodies used	Details of antibodies including their concentration are provided in Supplementary Table 1.
Validation	Antibody validation was multi-tiered: First, antibodies were selected based on their performance according to the manufacturer website and published literature. Their pattern of expression was then evaluated by secondary immunofluorescence staining of diverse tissues e.g. breast, colon, tonsil and lymph node. Antibodies showing staining patterns consistent with known biology were conjugated to metal isotopes and data acquired by imaging mass cytometry. Where the pattern of staining was preserved, multiplexed experiments were conducted to determine whether patterns of co-expression were consistent with known biology. Antibodies were titrated using human breast tumours to identify concentrations that best preserved both sensitivity and specificity. Supplementary Table 1 contains details of antibody registry ids (https://antibodyregistry.org/) which includes references to past applications where applicable.

Human research participants

Policy information about studies involving human research participants

Population characteristics	The study was an observational case-series of women with early breast cancer to conduct molecular profiling of primary tumours. Tumour samples, all surgically excised prior to systemic therapy, from 693 patients were used (mean age 62 years; range 22 to 96). Sample size was determined by whether suitable FFPE tissue was available for research; there were no exclusion criteria.
Recruitment	Participants were recruited while undergoing standard treatment at participating centres. There was no cause of self-selection bias.

Ethics oversight	University of Cambridge (REC ref 07/H0308/161)

Note that full information on the approval of the study protocol must also be provided in the manuscript.
